# RUNX3 regulates hepatocellular carcinoma cell metastasis via targeting miR-186/E-cadherin/EMT pathway

**DOI:** 10.18632/oncotarget.18424

**Published:** 2017-06-09

**Authors:** Yuli Gou, Fangbing Zhai, Liang Zhang, Lan Cui

**Affiliations:** ^1^ Department of Hepatobiliary Surgery, The Second Affiliated Hospital of Dalian Medical University, Dalian 116027, Liaoning, China; ^2^ Department of Radiology, The Second Affiliated Hospital of Dalian Medical University, Dalian 116027, Liaoning, China; ^3^ Department of Interventional Therapy, The Second Affiliated Hospital of Dalian Medical University, Dalian 116027, Liaoning, China; ^4^ Department of Ophthalmology, The Second Affiliated Hospital of Dalian Medical University, Dalian 116027, Liaoning, China

**Keywords:** RUNX3, miR-186, E-cadherin, HCC

## Abstract

Runt-related transcription factor 3 (RUNX3) has been reported as a tumor suppressor in some kinds of cancers. In the present study, hepatocellular carcinoma (HCC) microarray analysis showed that RUNX3 expression was significantly lower in HCC tissues compared with that in adjacent non-tumor tissues, and was negatively associated with metastasis and TNM stage. RUNX3 was an independently prognostic factor for 5-year overall and disease-free patient survival. Mechanically, RUNX3 repressed metastasis and invasion of HCC, and increased E-cadherin expression. RUNX3 also repressed microRNA-186 to increase E-cadherin expression. We demonstrated that miR-186 mimics attenuated RUNX3-induced increase of E-cadherin and inhibition of metastasis and invasion. In conclusion, RUNX3 suppressed HCC cell migration and invasion by targeting the miR-186/E-cadherin/EMT pathway. RUNX3 may be recommended as an effective prognostic indicator and therapeutic target for patients with HCC.

## INTRODUCTION

Hepatocarcinoma is the leading cause of cancer-associated death all over the world [1, 2]. Treatment outcomes for patients with hepatocarcinoma remain poor in spite of substantial advancements. Currently, the 5-year survival rate accounts for approximately 15% for hepatocarcinoma patients [3]. The metastatic tendencies, resistance to different treatments (such as targeted therapy, chemotherapy, radiotherapy), diagnosis at advanced stages and failure to respond to therapy predominantly contribute to the low survival rate [4]. Therefore, it is essential to find novel strategies to minimize the hepatocarcinoma metastasis.

RUNX3 is a member of Runt domain family members, and is localized on chromosome 1p.13-p36.11, and has an evolutionarily conserved 128 amino-acid domains [5]. RUNX3 binds to promoters or enhancers of genes to modulate gene expression involved in cell proliferation and differentiation [6, 7]. RUNX3 expression is often found in many types of cancers, and plays a tumor suppressor role in cancer development [8]. RUNX3 contributes to tumor initiation and metastasis in different biological behaviors, including EMT [9, 10], proliferation [11], migration and invasion [12]. However, the mechanisms of RUNX3 tumorigenesis and metastasis promotion are as yet unclear. Besides, E-cadherin expression is decreased in EMT of some cancers [13, 14]. In addition, miRNAs also regulate EMT by regulating related target genes [15, 16]. However, the role of RUNX3 in the miR-186/E-cadherin/EMT pathway was not elucidated.

In the present study, we investigated the relationship between RUNX3 expression and clinicopathologic features, *in vitro* and *in vivo* experiments were used to investigate the role of RUNX3 in HCC metastasis and E-cadherin expression. Moreover, we explored whether miR-186 overexpression affected RUNX3-induced E-cadherin increase and repression of HCC metastasis. This study will provide a potential therapeutic target for HCC patients.

## RESULTS

### RUNX3 expression is decreased in human HCC tissues

**Figure 1:**
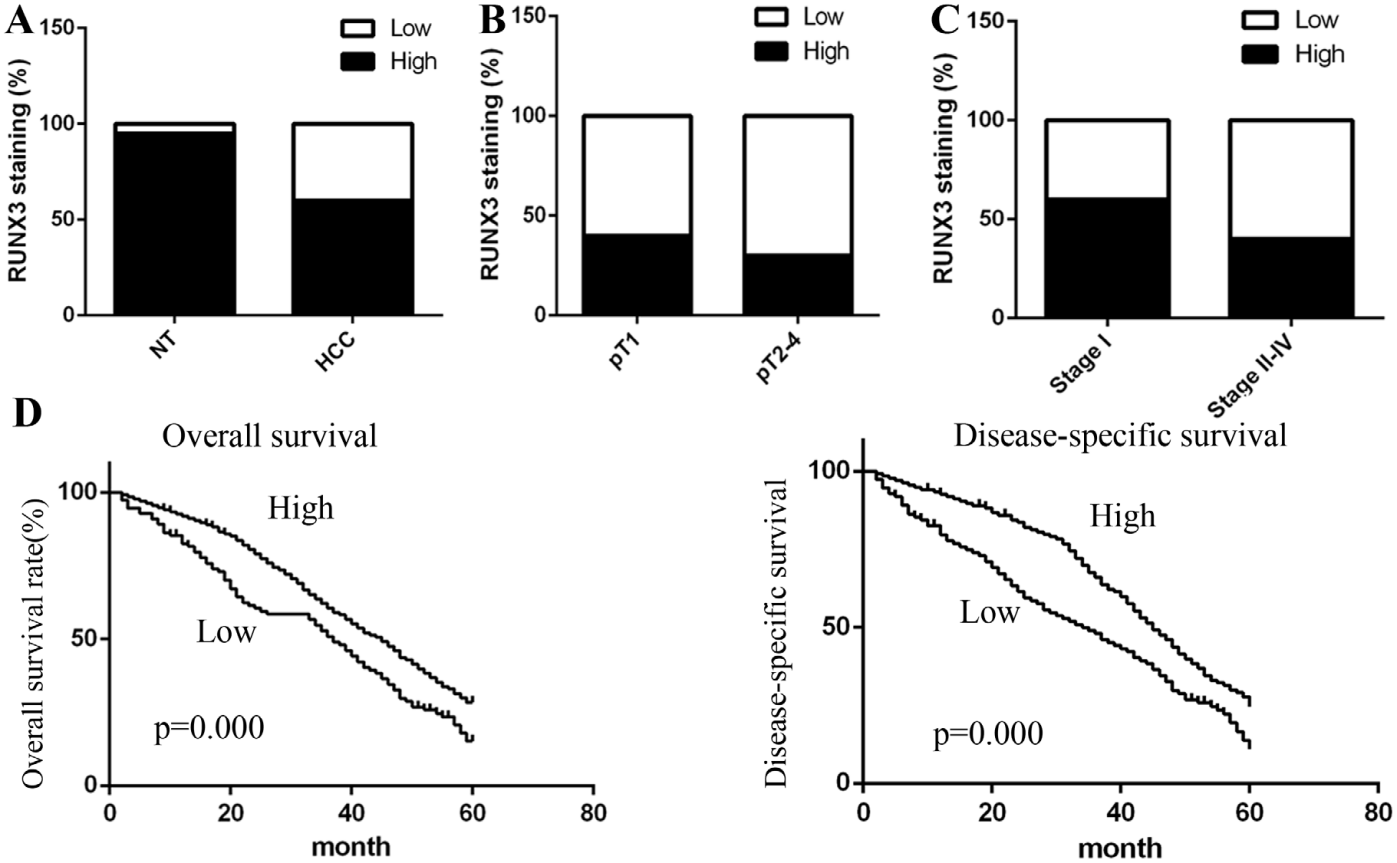
Correlation between RUNX3 expression and HCC clinicopathologic parameters Low RUNX3 expression was correlated with depth of invasion **(A)** (P<0.001, χ2 test, comparing pT1 versus pT2–pT4) and TNM stage **(B-C)** (P<0.001, c2 test, comparing I versus II-IV). **(D)** Kaplan–Meier curves of 5-year OS (P<0.001, log rank test) and DSS (P <0.001, log rank test).

Firstly, we used immunohistochemical (IHC) staining RUNX3 expression in cancer tissues and matched non-tumor tissues. We found that RUNX3 staining was mainly situated in the cytoplasm and nucleus of cancer cells in matched non-tumor tissues, while RUNX3 staining level was decreased in cancer tissues compared with matched non-tumor tissues (P <0.001, Figure 1A-1B), indicating that RUNX3 was involved in the development of HCC.

### Association between RUNX3 expression and clinicopathologic indicators

As shown in Table 1, we summarized the clinicopathologic parameters of 300 HCC patients. The expression of RUNX3 was inversely associated with depth of invasion (pT status; pT1 versus pT2-pT4) (P<0.001, Figure 1) and TNM stages (P<0.001, Figure 1). However, we observed no obvious correlations between RUNX3 and patient gender, age, tumor volume, or grade (Table 1).

**Table 1:** RUNX3 and clinicopathological characteristics of HCC patients

Variables	RUNX3		*P* value
	Low (%)	High (%)	
Age			
≤50 years	66 (46.5)	76 (53.5)	0.398
>50 years	65 (41.1)	93 (58.9)	
Gender			
Male	86 (42.6)	116 (57.4)	0.422
Female	47 (48.0)	53 (52.0)	
Tumor size			
≤5 cm	98 (42.6)	132 (57.4)	0.621
>5 cm	35 (50.0)	35 (50.0)	
Grade			
I+II	59 (38.1)	96 (61.9)	0.699
III+ IV	59 (40.7)	86 (59.3)	
TNM stage			
I+II	92 (42.4)	125 (57.6)	<0.001
III+ IV	32 (65.3)	17 (34.6)	

### Association between RUNX3 and patient survival

We employed overall survival (OS) and disease-specific survival (DSS) to analyze the role of RUNX3 in patient prognosis. 243 overall survival events and 214 disease-specific mortality events were summarized in the present study. Kaplan-Meier survival analyses showed that patients with RUNX3-negative tumors has a poor 5-year overall (P<0.001, log rank test) and disease-specific (P<0.001, log rank test) survival compared with those with RUNX3-positive patients (Figure 1D). Next, we used univariate and multivariate Cox regression model to determine significant factors, including RUNX3 expression, tumor volume, age, and TNM stage. Multivariate Cox regression analyses identified that RUNX3 was an independent prognostic biomarker for HCC patient OS (hazard ratio 0.339, P=0.001) and DSS (hazard ratio 0.198, P<0.000) (Table 2). Our findings validated that RUNX3 expression was obviously correlated with poor prognosis.

**Table 2:** Multivariate Cox regression analysis on 5-year overall and disease-specific survival of HCC patients

Variable*	Overall survival			Disease-specific survival		
	Hazard ratio	95% CI†	*P*	Hazard ratio	95% CI	*P*
RUNX3	0.339	0.145 to 0.611	0.001	0.198	0.098 to 0.298	0.000
Age	0.899	0.535 to 1.833	0.879	0.767	0.455 to 1.688	0.821
Tumor size	1.512	1.009 to 2.004	0.046	1.588	1.123 to 2.178	0.012
TNM stage	1.778	1.476 to 3.135	0.021	1.819	1.114 to 2.122	0.011

### RUNX3 negatively regulates HCC cell migration and invasion *in vitro*

**Figure 2:**
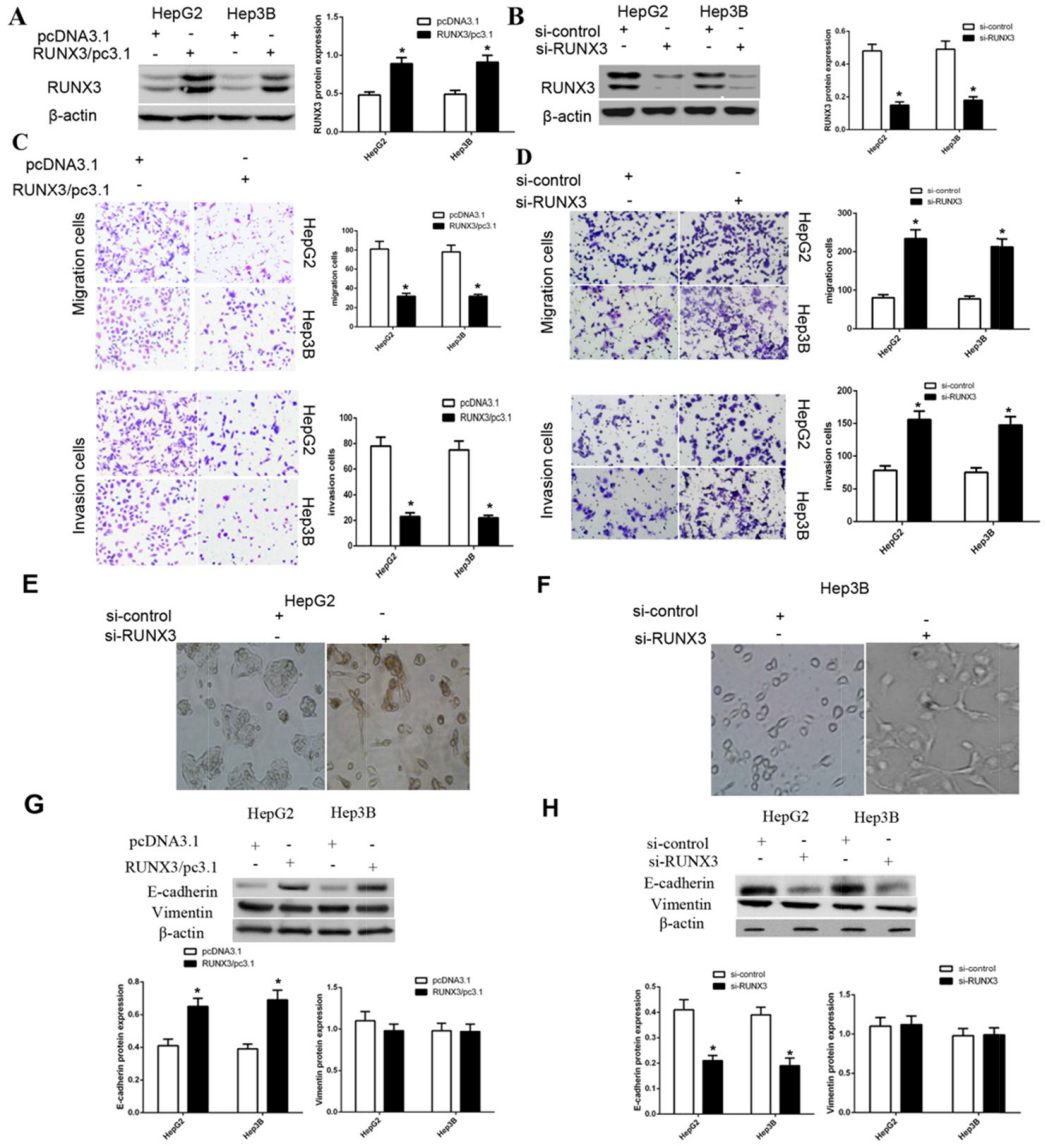
RUNX3 negatively regulates hepatocellular carcinoma cell motility and activates E-cadherin expression **(A-B)** Western blotting showed that 24 or 48 h after transfection, RUNX3 was overexpressed or decreased as expected in hepatocellular carcinoma cells. β-actin was used as an internal control. **(C-D)** RUNX3 overexpression inhibited migration and invasion in HepG2 and Hep3B cells; RUNX3 knockdown promoted migration and invasion. **(E-F)** EMT-like morphological changes in HepG2 and Hep3B cells transfected with control siRNA or RUNX3 siRNA. Cellular morphology was examined under light microscopy after 48 h. **(G-H)** Western blot analyses for E-cadherin and Vimentin in HepG2 and Hep3B cells transfected with pcDNA3.1 or RUNX3/pc3.1 or control siRNA or RUNX3 siRNA. All experiments were performed in triplicate. Data are shown as means ± SD (*P<0.05).

To elucidate the role of RUNX3 in cancer cells, we transfected RUNX3 plasmids or vector control, RUNX3 siRNA or control siRNA into HepG2 and Hep3B cells. Western blot analysis showed that RUNX3 expression was obviously up-regulated or downregulated after post-transfection 24 or 48 h (Figure 2A-2B). Function studies identified that RUNX3 overexpression repressed cell migration and invasion in cancer cells. By contrast, inhibition of RUNX3 expression promoted cell migration and invasion (Figure 2C-2D).

### RUNX3 upregulates E-cadherin expression

As shown in Figure 2E-2F, RUNX3 silencing altered cell morphology of HCC cells from a paving stone-like shape to a fibroblast-like spindle shape. Meanwhile, well-constructed cell-cell adhesion and mesenchymal cell phenotypes were lost. Since EMT was characterized by cancer cell migration and invasion, we detected the expression level of E-cadherin and Vimentin in cells with control or RUNX3 plasmids or control siRNA or RUNX3 siRNA. We found that RUNX3 positively modulated the expression of E-cadherin, while RUNX3 did not changed the expression of Vimentin (Figure 2G-2H).

### RUNX3 affects metastasis in nude mice model

**Figure 3:**
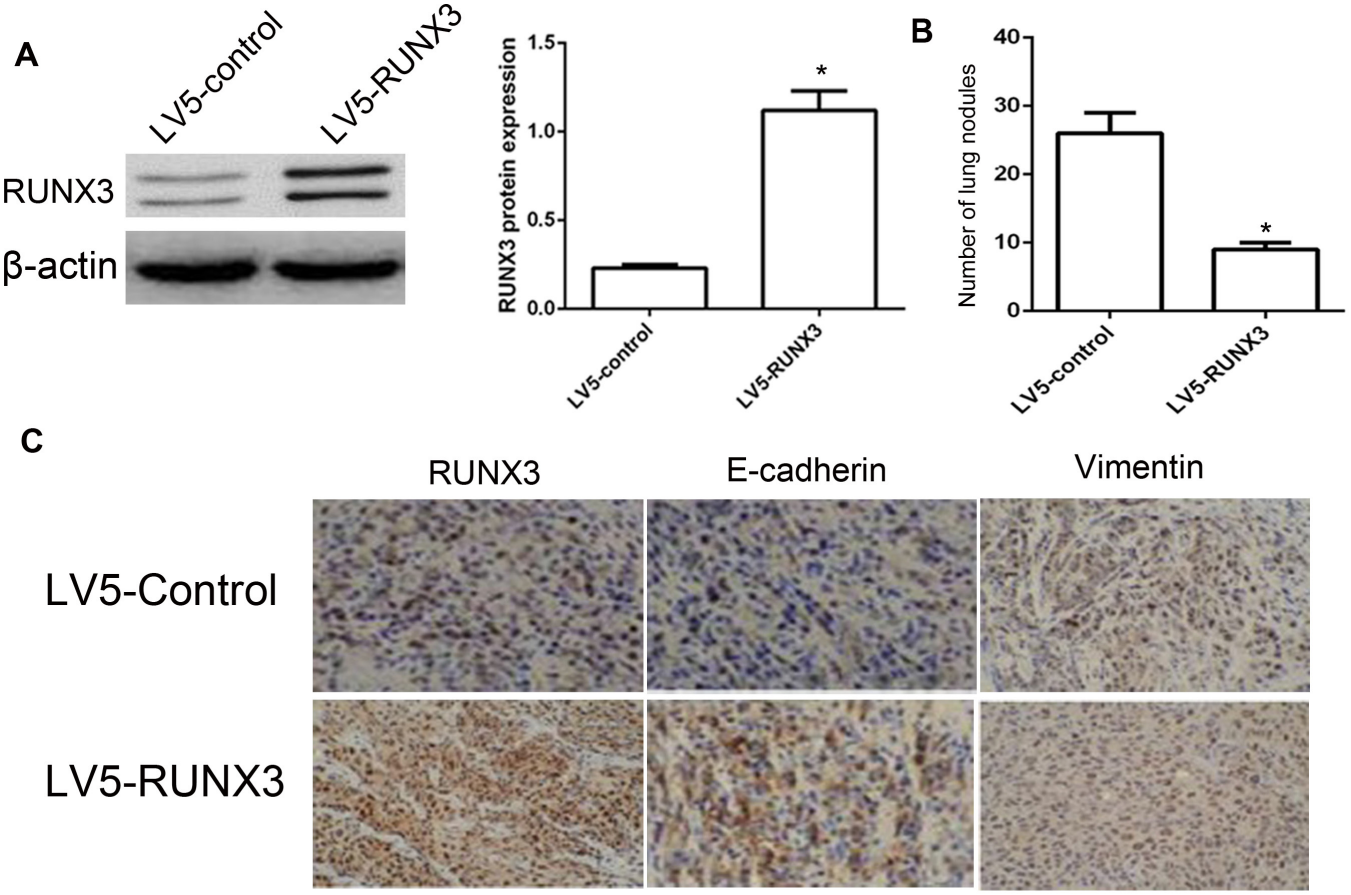
RUNX3 suppressed HCC metastasis *in vivo* Nude mice were injected intravenously with HepG2 cells stably expressing LV5-RUNX3 or LV5-Control. Western blot was used to examine RUNX3 level **(A)** Representative image of lung with metastatic nodules (top) and H&E staining (middle ×100, bottom ×400) 2 months after injection **(B)** Fewer lung metastases were seen in the LV5-RUNX3 group compared with controls. Data are shown with means ± SD (*P<0.05). **(C)** Immunostaining of RUNX3, E-cadherin and Vimentin in metastatic nodules of LV5-RUNX3 or LV5-Control groups.

Firstly, we demonstrated that RUNX3 can be stably and highly expressed in HepG2 cells (Figure 3A). We injected HepG2 cells infected with LV5-Control or LV5-RUNX3 into nude mice via their tail veins. And then, mice were euthanized after post-implantation 60 days, and surgically removed and fixed their lungs with 10% formal dehyde, and counts metastatic nodule number. We observed that lungs in LV5-RUNX3 nude mice had fewer and smaller detectable tumor nodules than control, indicating that RUNX3 reduces metastasis in nude mice model (Figure 3B). Next, we detected the expression of E-cadherin and Vimentin in metastatic nodule tissues of lungs. IHC analysis revealed that the expression of E-cadherin in the LV5-RUNX3 group was much higher compared with control. As expect, the expression of Vimentin was not changed (Figure 3C).

### RUNX3 directly binds the promoter of miR-186

**Figure 4:**
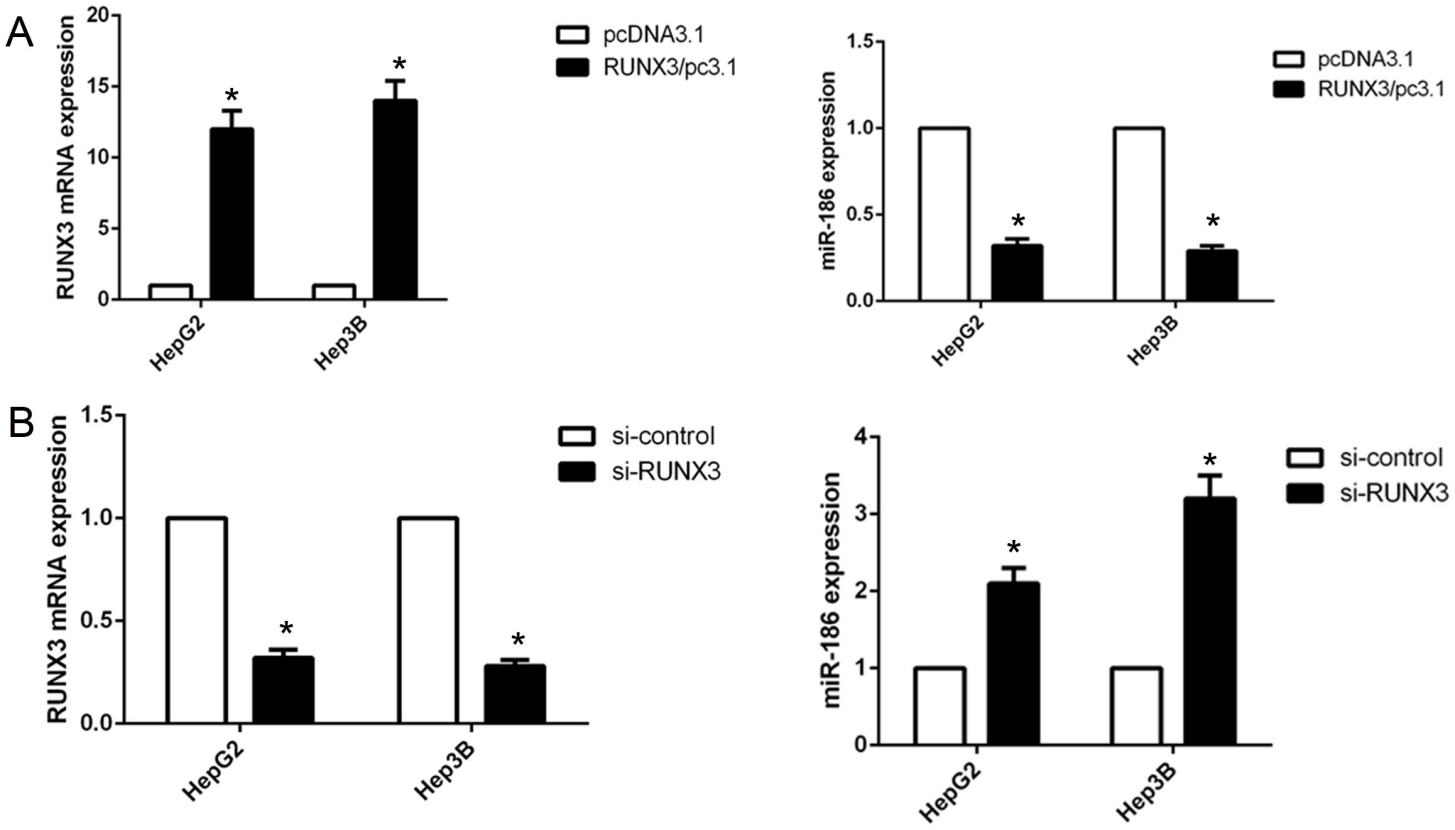
RUNX3 negatively regulated miR-186 expression in hepatocellular carcinoma cells **(A-B)** qRT-PCR analysis of RUNX3 and miR-186 levels in HepG2 and Hep3B cells transfected with RUNX3 overexpression plasmid or RUNX3 siRNA. Data were means ±SD (*P<0.05) from three experiments.

miRNAs have been identified to play an important role in the EMT processes, we applied a miRNA array to detected the miRNA profiles of HepG2 RUNX3 knockdown and control cells. We observed that 43 kinds of miRNAs were increased by over two-fold in HepG2 RUNX3 knockdown cells. And then we utilized the common algorithms to identify differentially expressed miRNAs, and validated that miR-186 could well bind to the conserved sites of E-cadherin 3’-UTR. So, we detect miR-186 expression in HCC cancer cells HepG2 and Hep3B with overexpressed or knocked down RUNX3 using qRT-PCR. Finally, we demonstrated that RUNX3 overexpression increased the expression of miR-186, while RUNX3 knockdown decreased the expression of miR-186 (Figure 4A-4B). Next, we analyzed the upstream region of miR-186 and identified one RUNX3 consensus binding sequences. We employed chromatin immunoprecipitation (ChIP), and found that inhibition of RUNX3 expression in HCC cancer cells reduced immunoprecipitated DNA from the miR-186 promoter, indicating that RUNX3 was able to bind the promoter of miR-186.

### miR-186 downregulates E-cadherin by binding the E-cadherin 3’ UTR

**Figure 5:**
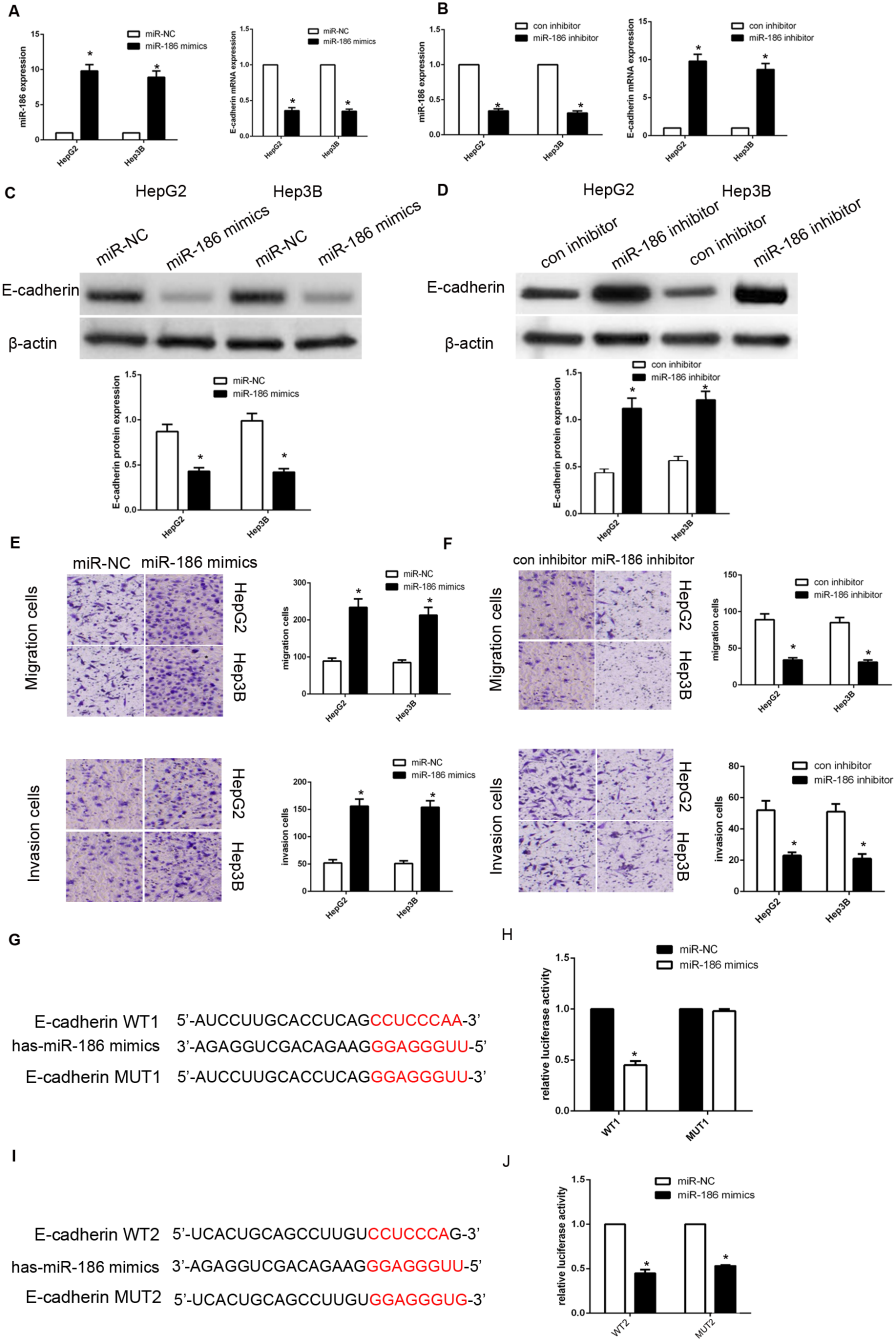
MiR-186 downregulated E-cadherin expression by directly targeting its 3’-UTR **(A-D)** qRT-PCR and western blotting for E-cadherin in HepG2 and Hep3B cells transfected with miR-186 mimics or inhibitor. **(E)** MiR-186 overexpression promoted migration and invasion in HepG2 and Hep3B cells. **(F)** miR-186 knockdown inhibited migration and invasion. **(G, I)** The two predicted sites of miR-186 binding to the E-cadherin 3’-UTR were detected using bioinformatics tools. **(H, J)** Mutated sites in the E-cadherin 3’-UTR are shown. The effect of miR-186 on luciferase activity induced by the psiCHECK™-2-E-cadherin-WT1, psiCHECK™-2-E-cadherin-mMUT1, psiCHECK™-2-E-cadherin-WT2 and psiCHECK™-2-E-cadherin-MUT2 reporter plasmids in HepG2 cells. Data are shown as means ± S.D. of three replicates (*P<0.05).

We used western blot to investigate the effect of miR-186 on endogenous E-cadherin expression. Here, Transfection of miR-186 mimics into HepG2 or Hep3B cells obviously decreased the expression of E-cadherin and promoted cancer cells metastasis. On the other hand, miR-186 inhibitor promoted the expression of E-cadherin (Figure 5A-5D) and repressed cancer cell migration and invasion (Figure 5E-5F). To validate whether miR-186 targets E-cadherin mRNA, the 3’-UTR of E-cadherin was cloned into the psiCHECK™-2 vector. We found that miR-186 mimics repressed luciferase activity, indicating that miR-186 inhibits 3’-UTR of E-cadherin. And then we constructed the mutated (mutant type, MUT) reporter at miR-186 binding site. We found that the activity of MUT1 3’-UTR reporter was not altered (Figure 5G-5J). These findings showed that miR-186 could directly target the 3’-UTR of E-cadherin and downregulated E-cadherin expression.

### miR-186 mimics abrogates RUNX3-induced E-cadherin expression and metastasis suppression

**Figure 6:**
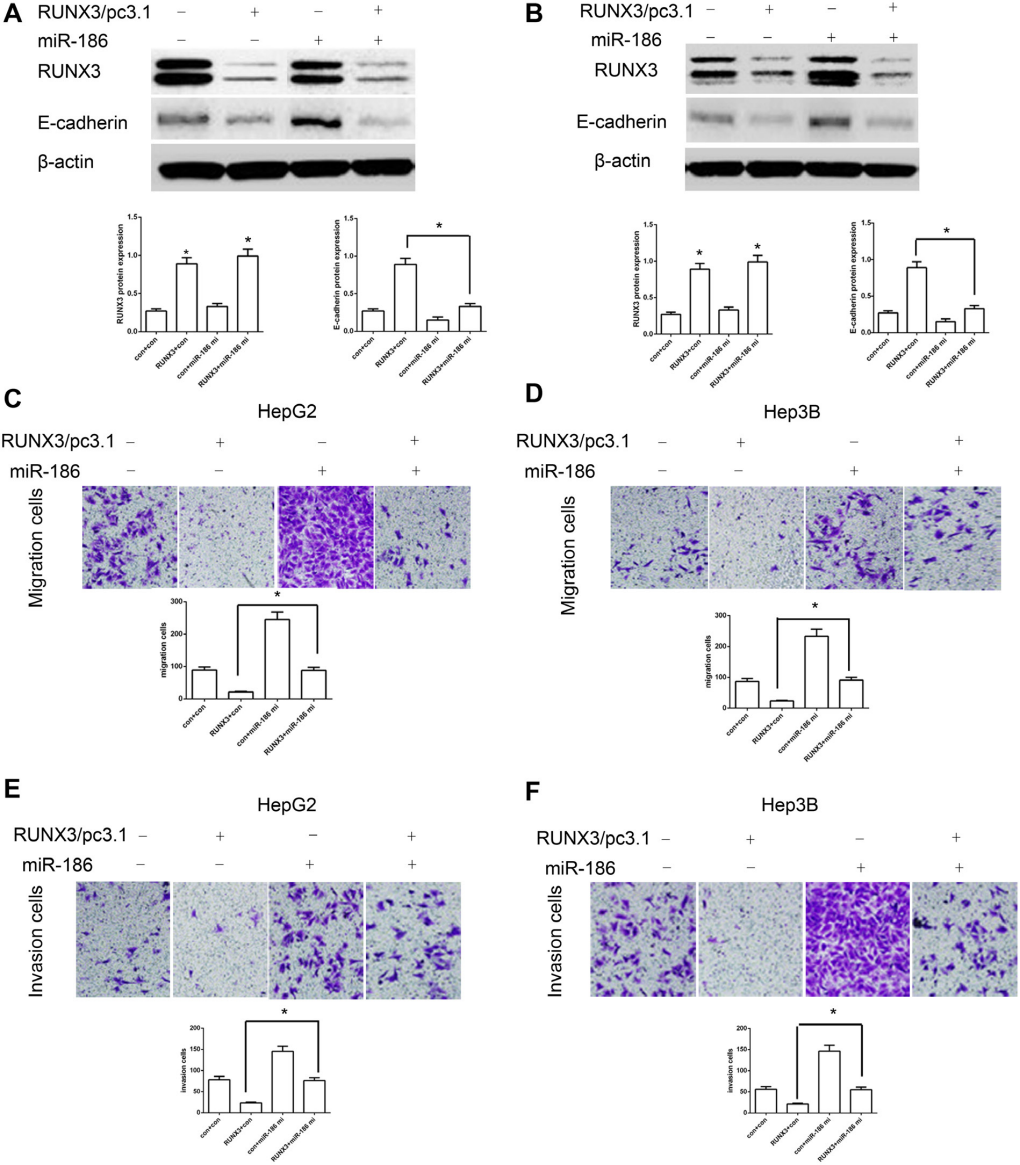
MiR-186 mimics abrogated RUNX3-mediated E-cadherin upregulation and HCC metastasis inhibition **(A-B)** Western blot analysis showed that miR-186 mimics blocked RUNX3-mediated E-cadherin upregulation in HepG2 and Hep3B cells. **(C-F)** Cell migration and invasion assays in HepG2 and Hep3B cells infected by control, RUNX3/pc3.1, miR-186 mimics or RUNX3/pc3.1 with miR-186 mimics. Migration and invasion were greater in the RUNX3/pc3.1 plus miR-186 mimics group compared with the RUNX3/pc3.1 group. Data are shown as means ±SD of three replicates (*P<0.05).

To identify whether miR-186 controlled RUNX3-induced cancer metastasis inhibition, we miR-186 mimics to up-regulate the expression of miR-186 in RUNX3-overexpressing cells. Compared with control, ectopic miR-186 expression attenuated RUNX3-induced the expression of E-cadherin (Figure 6A-6B) and abrogated RUNX3-mediated cell migration and invasion inhibition (Figure 6C-6F). These findings indicated that RUNX3-mediated E-cadherin expression and metastasis inhibition largely depend on miR-186 inhibition.

## DISCUSSION

In recent years, the effect of RUNX3 on oncogenesis and metastasis has been widely reported [20]. RUNX3 has been identified to play multiple tumor suppressor roles in many kinds of human cancers, including gastric cancer, colorectal cancer, and breast cancer [11, 21–24]. Hypermethylation of CpG Island and point mutations were responsible for decreased RUNX3 expression [25]. However, the involvement of RUNX3 in development and metastasis of HCC was completely unknown. In this work, we investigated whether RUNX3 induces tumorigenesis and metastasis in HCC.

In the present study, RUNX3 expression was decreased in HCC tumor tissues compared with normal hepatocellular tissues. We further assessed correlations between RUNX3 expression and clinicopathological features, and verified that RUNX3 decrease was correlated with metastasis and TNM stage. Cox proportional hazards regression suggested that RUNX3 was negatively correlated with poor OS and DSS of HCC patients. Therefore, RUNX3 might play an important role in the progression of HCC and may predict the prognosis of patients with HCC.

As known to all, when the expression of tumor suppressive genes was decreased, cell motility and invasion will be primed [26]. Our findings revealed that depletion of RUNX3 promoted HCC cell migration and invasion into matrigel-coated chambers, while overexpression of RUNX3 repressed cell migration and invasion, indicating that RUNX3 has the potential to modulate HCC metastasis. As reported, EMT is a critical process for malignant change of cancers, especially metastasis [27, 28]. So, we further detected the expression of E-cadherin and Vimentin in HCC cells with RUNX3 overexpression or depletion. We found that RUNX3 positively modulates the expression of E-cadherin, however Vimentin expression was not altered. According to recent reports, ectopic expression of RUNX3 promoted E-cadherin expression, but had a negative effect on Vimentin in hepatocellular carcinoma cells [29]. In addition, Liu et al indicated RUNX3 suppresses the expression of Vimentin instead of E-cadherin expression in gastric cancer [30–33]. Our findings indicated that RUNX3 overexpression could not repress the expression of Vimentin, but induce the expression of E-cadherin of HCC cells.

MicroRNAs are a kind of small, non-coding, single-strand RNAs, which consist of 20-22 nucleotides (nt), and have been identified as a potential regulator of EMT [34, 35]. miRNAs regulated the expression of genes by binding their own untranslated regions, leading to degradation of the target mRNAs or inhibition of translation [36]. As reported, miR-9 can target E-cadherin-encoding messenger RNA and mediate a mesenchymal phenotype to promote migration or invasion capacity [37]. So, we assumed that RUNX3 may regulate the expression of miRNA to downregulate E-cadherin in HCC. We found that RUNX3 directly binds to the miR-186 promoter and repressed expression of the miR-186. Consistent with our study, RUNX3 could directly bind the promoter of miR-30a and promotes the expression of miR-30a [38]. Furthermore, miR-186 overexpression decreased the expression of E-cadherin and induces metastasis in human HCC cells. By contrast, inhibition of miR-186 promoted E-cadherin expression and repressed metastasis. Besides, miR-186 mimics abrogated RUNX3-induced increase of E-cadherin and inhibition of metastasis. These results indicated that RUNX3 upregulated E-cadherin and repressed metastasis by downregulating miR-186 expression.

In conclusion, our work identified that RUNX3 downregulation is involved into tumorigenesis and metastasis of HCC. Mechanically, RUNX3 negatively modulates miR-186 expression by directly binding miR-186 promoter, and miR-186 downregulates E-cadherin expression by directly binding its 3’UTR. We may provide a novel therapeutic target for HCC patients.

## MATERIALS AND METHODS

### Patient samples

The study material includes 300 HCC and 35 adjacent normal hepatocellular tissues cases from The Second Affiliated Hospital of Dalian Medical University, treated between 2014 and 2015. All patients underwent surgery only or postoperative adjuvant therapy. The patients’ clinicopathologic information was obtained from the pathology department archive and was confirmed by hospital medical records. Follow-up information was obtained by reviewing patient medical records.

### Antibodies and reagents

Anti-RUNX3 mouse monoclonal antibodies were purchased from MBL (Medical and Biological Laboratories, Nagoya, Japan). Rabbit monoclonal antibodies to E-cadherin and Vimentin were purchased from BD Biosciences (NJ, USA). Mouse anti-β-actin was from Boster Biotechnology (Wuhan, China). Rabbit antibodies to TWIST1 and SNAI2 were purchased from BIOSYNTHESIS (Beijing, China).

### Immunohistochemistry

IHC staining was performed as described previously [17]. The primary mouse anti-RUNX3 antibody (1:100), anti-E-cadherin and Vimentin (1:100) were used. For TMA staining evaluation, immunoreactivity was assessed by two blinded independent observers using light microscopy (Olympus BX-51 light microscope), and images were collected by a Camedia Master C-3040 digital camera. RUNX3 expression was graded as positive when 10% of tumor cells showed immunopositivity. Biopsies with less than 10% tumor cells showing immunostaining were considered negative. The detailed conditions are described previously [18].

### Animals and cell lines

Female BALB/c nude mice, 6 weeks old, were purchased from the Shanghai Laboratory Animal Center (Shanghai, China) for studies approved by the Animal Care Committee of Xuzhou Medical College. Human HCC cell lines HepG2 and Hep3B were purchased from the Shanghai Institute of Biochemistry and Cell Biology, Chinese Academy of Sciences (Shanghai, China). HepG2 and Hep3B cells were cultured in RPMI1640 medium supplemented with 10% fetal calf serum (Invitrogen, Shanghai, China). Cells were in a 37°C humidified incubator with 95% air, 5% CO_2_.

### Transfection

pcDNA3.1 and RUNX3/pc3.1 expression plasmids, non-specific control siRNA or RUNX3 siRNA were purchased from Integrated Biotech Solutions (Shanghai, China). Transient transfection of plasmids into hepatocellular carcinoma cells was carried out using Lipofectamine 2000 transfection reagent (Invitrogen, Shanghai, China) following the manufacturer’s protocol. The sequences for the RUNX3 siRNA and control siRNA were 5’-CCUUCAAGGUGGUGGCAUUTT-3’ and 5’-UUCUCCGAACGUGUCACGUTT-3’, respectively. Cells were transfected with siRNA using siLentFect Lipid Reagent (Bio-Rad, Hercules, CA, USA) according to the manufacturer’s instructions. Human miR-186 mimics, control mimics, inhibitors and control inhibitors were synthesized by Integrated Biotech Solutions (Shanghai, China).

For stable transfection, the lentiviral expression vectors LV5-Control and LV5-RUNX3 were obtained from Shanghai Gene Pharma Company (China). Lentiviruses were mixed with polybrene (5 mg/ml) and added HepG2 cells. Positive clones were selected in puromycin (5 mg/ml). Stable RUNX3 transfectants were isolated after 2 weeks.

### Cell migration and invasion assays

Cell migration and invasion assays were performed using modified two chamber plates with a pore size of 8 μm. Transwell filter inserts with or without Matrigel (BD Biosciences) coating were used, respectively, for invasion and migration assays. Detailed conditions were described previously [19].

### Western blot analysis

Cell extracts were separated on a 10% SDS-polyacrylamide gel and proteins were transferred to nitrocellulose membranes and incubated overnight at 4°C with the following antibodies: mouse anti-RUNX3, β-actin, rabbit anti-E-cadherin, Vimentin, SNAI2 or TWIST1. After incubation with peroxidase-coupled anti-mouse or anti-rabbit IgG at 37°C for 2 hours, membranes were washed and scanned using the Odyssey Two-Color Infrared Imaging System (LI-COR Biotechnology, Lincoln, Nebraska, USA). Each western blot was repeated three times.

### RNA extraction and real-time PCR

RNA extraction was carried out using Trizol (Invitrogen) according to the manufacturer’s instructions. Real-time PCR amplification of RUNX3, E-cadherin and GAPDH was performed for 30 s at 95°C, followed by 40 cycles at 95°C for 5 s and annealing at 60°C for 34 s using an ABI PRISM 7500 Sequence Detection System (New York, USA). Target mRNA levels were calculated based on the CT method, normalized to GAPDH, and are expressed as a ratio of the percentage of gene copies to the GAPDH control. All reactions were run in triplicate. The primer sequences for each gene are as follows: RUNX3 Forward, 5’-TACCTGGCAAGTCCCTCCCGAACA-3’, Reverse, 5’-AGGCCTATATGGGTCCAAACTT-3’; E-cadherin Forward, 5’-TTCCTCCCAATACATCT CCC-3’, Reverse, 5’-TAACCGTTAGTAGTCACCCACC-3’; GAPDH Forward, 5’-ACCGCAATTGGGAGTCAGGATT-3’, Reverse, 5’-CCAATGTGTCCAGGAGGATT-3’.

### Reporter vector construction and luciferase assay

The 204-bp miR-186 binding sequence at the human E-cadherin gene 3’ UTR was cloned into the XhoI and NotI restrictions sites downstream of the Renilla luciferase gene in the psiCHECK™-2 vector. Mutagenesis of the putative miR-186 binding sites was performed using the QuikChange site directed mutagenesis kit. Primer sequences are listed in Figure 5. Cells were seeded in 48-well plates and transiently transfected with appropriate reporter plasmids and miRNA using Lipofectamine 2000. Cells were harvested and lysed after 48 h. Luciferase activity was measured using the Dual-Luciferase Reporter Assay System (Promega, Madison, WI, USA), normalized to renilla luciferase. For each plasmid construct, transfection experiments were performed in triplicate.

### Chromatin immunoprecipitation

HepG2 or Hep3B cells transfected with control siRNA or RUNX3 siRNA were cross-linked by incubation in 1% (v/v) formaldehyde-containing medium for 10 min at 37°C, then sonicated to form soluble chromatin. A RUNX3 antibody was used to precipitate DNA fragments bound by their corresponding elements. The protein–DNA complex was collected using protein A Sepharose beads (Millipore) and then eluted and reverse cross-linked. After protease K treatment, samples were extracted with phenol/chloroform and precipitated with ethanol. Recovered DNA was re-suspended in TE buffer and amplified by PCR. Primers used for ChIP assay were as follows: forward 5’-CACCTGTGAATTCTCTGACC-3’ and reverse 5’-TTCCGAATGCCCTAAGTTAG-3’, and control primers for GAPDH.

### Mouse model

BALB/c nude mice were randomly divided into two groups of 8 mice each. LV5-Control and LV5-RUNX3 HepG2 cells were suspended in PBS. Mice were injected intravenously with 2.5×10^6^ HepG2 cells in 0.2 ml of PBS via tail vein. After 2 months, mice were sacrificed and their lungs were resected and fixed in 10% buffered formalin. The number of metastatic nodules on the surface of each set of lungs was counted by visual inspection using a stereoscopic dissecting microscope.

### Statistical analysis

Data are expressed as means ± SD. Two-factor analysis of variance and the Dunnett’s t-test were used to assess differences within treatment groups. For TMA, statistical analysis was performed with SPSS 20 software (SPSS, Inc, Chicago, IL). Associations between RUNX3 staining and HCC patient clinicopathologic parameters, including age, gender, tumor size, grade, pT status and TNM stage, were evaluated by two-sided Fisher’s exact tests. The Kaplan-Meier method and log-rank test were used to evaluate the correlation between RUNX3 expression and patient survival. Hazard ratios (HR) and 95% confidence intervals (CI) were calculated using multivariate Cox proportional hazards regression models to analyze the independent impact of clinicopathologic factors and RUNX3 on survival. P<0.05 was considered statistically significant.
